# Supplementary material for: Complexome profiling of the Chlamydomonas psb28 mutant reveals TEF5 as an early PSII assembly factor
Source: Plant Cell. 2025 Mar 17;37(6):koaf055. doi: 10.1093/plcell/koaf055 (PMC12164590; doi:10.1093/plcell/koaf055)
Supplement: koaf055_Supplementary_Data [file koaf055_supplementary_data.zip › psb28_interactive.zip!/plots/intensity/qualityPlots/peptides/COQ5_Cre02.g084300.t1.2.html]

### Cre02.g084300.t1.2

Trivial name: COQ5  
SeqCoverage [median]: 14.40  
Mass: 31.52 kDa  
  
 

| Sequence | MissedCleavage | Mass [Da] | wtIntensity | muIntensity | Score |
| --- | --- | --- | --- | --- | --- |
| EAGFAAAR | 0 | 791.39 | 0.00 | 1602.10 | 82.39 |
| RFPTGPEQVR | 1 | 1185.63 | 748.86 | 699.44 | 62.63 |
| VATDDAALPGPQGAFVPGQEAEAR | 0 | 2366.15 | 798.49 | 1416.42 | 51.65 |
|  |  |  | 1547.4 | 3718.0 |  |
